# E-Cigarette Use Among Adolescents: An Overview of the Literature and Future Perspectives

**DOI:** 10.3389/fpubh.2018.00086

**Published:** 2018-03-26

**Authors:** Evanthia P. Perikleous, Paschalis Steiropoulos, Emmanouil Paraskakis, Theodoros C. Constantinidis, Evangelia Nena

**Affiliations:** ^1^Medical School, Democritus University of Thrace, Alexandroupolis, Greece; ^2^Department of Pneumonology, Medical School, Democritus University of Thrace, Alexandroupolis, Greece; ^3^Department of Pediatrics, Medical School, Democritus University of Thrace, Alexandroupolis, Greece; ^4^Laboratory of Hygiene and Environmental Protection, Medical School, Democritus University of Thrace, Alexandroupolis, Greece

**Keywords:** adolescents, teenagers, e-cigarettes, vaping, electronic cigarette

## Abstract

**Background:**

Electronic cigarettes (e-cigarettes) are rapidly emerging into a new trend among adolescents, signaling a new époque, that of vapers. E-cigarettes are battery-powered nicotine delivery devices that heat a typically flavoring liquid solution into an aerosol mist that users inhale, allowing them to imitate the act of conventional smoking. There are concerns about the impact of e-cigarettes at both individual and public health level.

**Aim:**

To discuss the characteristics of the most vulnerable, to become e-cigarette users, group of adolescents and to further highlight their behaviors and characteristics.

**Methods:**

An electronic search in PubMed, EMBASE, and Google Scholar databases was conducted, using combinations of the following keywords: adolescents, teenagers, e-cigarettes, vaping. The search included all types of articles written in English until August 2017. A total of 100 articles were found, and 25 were finally included in the present review.

**Results:**

Older age, male gender, conventional smokers, peer influence, daily smoking, and heavier smoking are the most common characteristics of adolescent e-cigarette users.

**Conclusion:**

E-cigarette use is common, especially between certain subgroups in the adolescent population. Since e-cigarette use is increasing and considering that the long term health effects are still under investigation, targeted interventions towards more susceptible individuals may be an effective prevention strategy.

## Introduction

### Rationale-Objectives

Tobacco purchase and usage have shifted to alternative products since the introduction of electronic nicotine delivery systems into the market in the mid-2000s, raising concerns due to increased public interest (1, 2). Electronic cigarettes (e-cigarettes) are novel battery-operated hand-held devices designed to deliver smokeless doses of nicotine, through a vaporization process. E-cigarettes have been designed to simulate the sensory experience of smoking, although without combustion.

Nowadays, a wide variety of e-cigarette brands is easily accessible in retail and online shops (3). E-cigarette advertising expenditures increased sharply (4), while safety and long-term health effects are still vague based on the present scientific evidence. As a result of the large-scale marketing, e-cigarettes gained widespread pervasiveness among all age groups, including vulnerable adolescents and youths populations (5–8). Indeed, recent reports from United States showed that 4.3% of middle school students and 11.3% of high-school students reported having used e-cigarettes in 2016 (9). In addition, reports from UK, comprising data from 60,000 young individuals, aged 11–16 years, showed regular e-cigarette use between 1 and 3% and ever-use between 7 and 18% (10). Furthermore, data from 24,658 individuals in the 2012 National Youth Tobacco Survey reported that almost one-third of adolescents in the United States consider e-cigarettes as less harmful than conventional cigarettes (11).

E-cigarette vapor contains many of the known harmful toxins of traditional cigarettes, such as formaldehyde, cadmium, and lead, even though usually at a reduced percentage (12). However, short- and long-term health implications on e-cigarette users remain foggy. E-cigarette marketing is of particular concern, because is creating an illusion that e-cigarettes are safer and healthier than conventional tobacco cigarettes, whereas their safety and their potential role in smoking cessation is still a matter of ongoing debate.

Diverse characteristics influence the vulnerability of adolescents toward e-cigarette usage. These can be intrapersonal, like adolescents’ age, interpersonal, like conflict with family and peers, and contextual comprising community structures and district laws (13). Several marketing and design product features seem to be more attractive for young people. For example, flavorings or lack of age regulation restricting laws have been implicated as reasons for youth susceptibility to e-cigarettes (14). An analysis of e-cigarette retail websites, marketing, and promotional campaigns demonstrated frequent appeals to adolescents such as use by celebrities, feature cartoons, and enhanced social activity as well as sexual appeal (15).

It is a common assumption that adolescents have higher rates of impulsivity, and therefore proclivity of adopting dangerous behaviors, rather than other age groups (16). According to the theoretical model of planned behavior, individuals’ perceptions influence their choice to participate in a specific behavior (17). Consistent with the aforementioned theory, many youths perceive e-cigarettes as safer, easier to conceal, and healthier alternatives compared with combustible cigarettes (18, 19). Youths who have lower harm perceptions may be particularly susceptible to e-cigarette and polytobacco use (11, 20–24), conversely those who perceive e-cigarettes as more harmful would be less possible to use them (11).

Marketing, especially through social media, has a salient role in vaping promotion among adolescents; whereas retail stores are a prominent source of e-cigarette display (25). Four Scottish communities participated in a recent observational study in which a potential concern has emerged due to the placement of e-cigarettes, in 36% of stores, near to products popular to children (26). E-cigarettes are often marketed and displayed on countertops near till points and next to products of particular interest to children and adolescents; this may lead to the embracing of e-cigarettes as a broadly used and accepted product (26). However, several US jurisdictions have passed laws that increased the minimum age of sale for all tobacco products, including e-cigarettes, to 21 years (27).

Future research is imperative to illustrate the motivations behind teenagers’ experimentation with e-cigarettes, while continued monitoring is warranted to clarify the temporal relationship between e-cigarette and tobacco products (28), with firmer tobacco control and social networking policies to prevent smoking initiation and lifetime continuation.

## Methods

### Search Strategy

**Figure 1:**
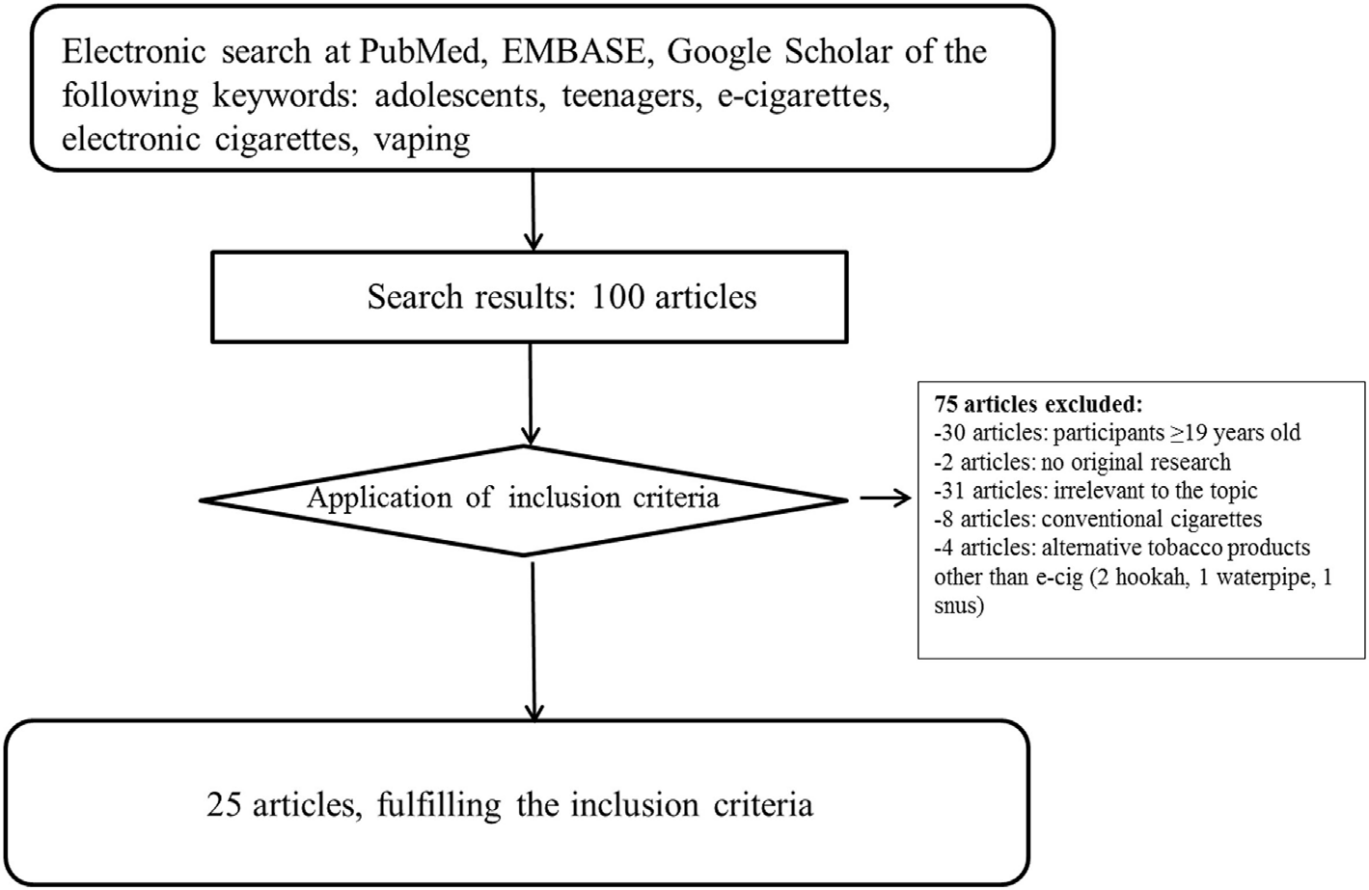
Flow chart of the search strategy.

We performed an electronic search in the following databases: PubMed, EMBASE, and Google Scholar, using combinations of the following keywords: adolescents, teenagers, e-cigarettes, vaping. The search included all types of articles written in English until August 2017. A total of 100 articles were found, and 25 were finally included in the present review. Exclusion criteria were the following: included participants older than 18 years (*n* = 30 articles), not original research (*n* = 2 articles), not relevant data, for example, e-cigarette marketing issues, consumers’ preference in certain products, etc. (*n* = 31 articles), use of conventional cigarettes (*n* = 8 articles), use of alternative tobacco products (*n* = 4). The followed strategy and search results are displayed in Figure 1.

## Results

The large body of evidence points to an increased interest in exploring the characteristics among adolescent e-cigarette users. Table 1 summarizes the current literature about the characteristics of adolescent users regarding e-cigarettes. E-cigarette has a large dispersion and penetration among teenagers, and is becoming the most commonly used tobacco product (9). Many researchers declared that the most frequent reason behind adolescents’ e-cigarette experimentation was curiosity and the irresistible urge to try something new (29–31). On the contrary, major causes of e-cigarette smoking cessation were the following: losing interest, perceiving them as uncool, and enunciating anxiety about health (14).

**Table 1:** Summary of studies assessing characteristics of adolescent electronic cigarettes users.

Study	Participants	Main results	Comments
Wang et al. (6)	– 24,658 middle and high-school students in United States – Assessment through interview	E-cigarette users [% (95% CI)] – Ever used conventional tobacco products [20.3 (18.3, 22.5)] – Currently use conventional tobacco products [12.9 (11.0, 15.0)] – Ever used other non-conventional tobacco products [34.0 (31.0, 37.2)] – Currently use other non-conventional tobacco products [19.0 (16.2, 22.2)]	– Almost two-thirds of participants were aware of one or more of e-cigarettes, hookah, snus, and dissolvables – Conventional tobacco users were more prone to use non-conventional tobacco products
Cooper et al. (20)	– 13,602 middle and high-school students in Texas – Assessment through a 38-item anonymous survey	– Non-users vs dual users harm perceptions about cigarettes; e-cigarettes; chew; snus; hookah; general tobacco; and alcohol all *P* < 0.001 – Non-users vs dual users peer use of cigarettes; e-cigarettes; and chew all *P* < 0.001	– Dual users were more likely to be white, male, and older – Non-users were significantly more likely to rate all tobacco products and alcohol use as more harmful compared with dual use group – Dual users perceived greater peer use of cigarettes compared with non-users
Kaleta et al. (21)	– 3,552 middle and high-school students in Poland – Assessment through anonymous, self-administered questionnaire	Current e-cigarette use was strongly associated with [OR (95% CI)] – Current [32.5 (23.2**–**45.5)]; *P* < 0.001 and ever tobacco smoking [7.5 (5.5**–**10.1)]; *P* < 0.001; smoking parents [1.4 (1.1**–**1.8)]; *P* < 0.05 and friends [4.5 (3.1–6.5)]; *P* < 0.05	– Male gender, alcohol users, current and ever tobacco smokers, parents’ and friends’ smoking were more likely to report current e-cigarette use – Use of e-cigarettes was significantly associated with harm perception (*P* < 0.001) – Higher paternal education and perception of e-cigarettes as more harmful compared with traditional cigarettes have a protective role in current e-cigarette use
Agaku et al. (22)	– 18,866 middle and high-school students in United States – Assessment through anonymous national survey	E-cigarettes users [OR (95% CI)] – Students exposed most of the time/always to retail [1.71 (1.21–2.41)] or Internet pro-tobacco advertisements [1.59 (1.17–2.16)] were more prone to use e-cigarettes – Tobacco use by at least one close friend [3.05 (2.17–4.28)] or family member [1.55 (1.17–2.07)], or being a current user of snuff, chewing tobacco, or dip [2.16 (1.61–2.91)], or of any combustible tobacco product [14.1 (10.57–18.82)] all increased the likelihood of experimenting with e-cigarettes	– Students who were exposed to retail or Internet pro-tobacco advertisements were more likely to use e-cigarettes – Tobacco use by close friends or household member, or being a current user of any combustible tobacco product increased the probability of experimenting with e-cigarettes
Giovacchini et al. (23)	– 947 middle school and high-school students in North Carolina – Assessment through anonymous, self-reported survey	– Harm perception of e-cigarette use decreased as grade level increased χ^2^ = 67.3, *P* < 0.001 – 49.4% of e-cigarette users had never smoked cigarettes. Ever-use of e-cigarettes was 37.2% and ever-use of combustible cigarettes was 21.7% – Compared with non-users, e-cigarette users were less likely to perceive e-cigarette use as having great risk (16.5% vs 3%; χ^2^ = 18.4, *P* < 0.001) – Friends’ harm perception of e-cigarette use [OR (95% CI)] – [0.43 (0.19**–**0.97)]	– Perception of great risk associated with e-cigarette use decreased with advancing grade – Ever-use of e-cigarettes surpassed ever-use of combustible cigarettes at all grades – Friends’ harm perception of e-cigarette use correlated negatively with e-cigarette use
Lee et al. (24)	– 24,658 middle and high-school students in United States – Assessment through national survey	– Among participants 6.7% used exclusively one product, 3.6% used two products, and 4.3% used ≥3 products – Polytobacco users were significantly associated with male gender (adjusted relative risk ratio = 3.71)	– Twice as many youth use exclusively e-cigarettes than dual use with cigarettes – Polytobacco use was associated with male gender – Authors postulated that e-cigarettes may be attractive to non-smoking youth and not likely used for cessation among smokers
Kinnunen et al. (29)	– 3,535 middle and high-school students in Finland – Assessment through self-administered questionnaires	E-cigarette ever-use [OR (95% CI)] – Parents’ high-educational level [1.0], parents’ middle educational level [1.78 (1.45–2.19)], parents’ low-educational level [1.74 (0.96–3.18)] – Fathers’ work situation – Working [1.0] – Not working [1.42 (1.09–1.83)] – Mother’s work situation – Working [1.0] – Not working [1.58 (1.21–2.07)] – Family structure – Intact family [1.0] – Other family type [1.73 (1.41–2.11)] – Daily smokers [120.86 (81.72–178.74)] – Ever-use of snus [12.05 (9.69–14.98)] – Ever-use of waterpipe [6.54 (5.27–8.12)] – Children’s vocational education [3.29 (2.60–4.17)] – Poor school performance [3.89 (2.96–5.12)] – Considered quitting smoking [% (95% CI)] – Use of e-cigarettes more than 20 times [55.3% (41.2–68.6)] – Daily e-cigarettes smokers, ≥10 cigarettes daily [48.6% (33.4–64.1)]	– Socioeconomic background such as parents’ high level of education, being in employment, and intact family protected against e-cigarette experimentation – Daily smoking, snus use, waterpipe use, male gender, children’s vocational education, and poor school performance were associated with e-cigarette use – Daily smokers of e-cigarettes were less likely to be interested in quitting smoking
Surís et al. (30)	– 621 high-school students in Switzerland – Assessment through an online, self-reported questionnaire	– 43% of participants had ever tried e-cigarettes, 19% were experimenters and 24% users – Compared with never users, experimenters were more likely to be – Out of school [relative risk ratio (RRR): 2.68] – Misuse alcohol (RRR: 2.08) – Users were more likely to be Male (RRR: 2.75) Vocational students (RRR: 2.30) Out of school (RRR: 3.48) To use tobacco (RRR: 5.26) To use alcohol (RRR: 2.71) To use cannabis (RRR: 30.2)	– Main reason to have ever tried e-cigarettes was curiosity – Compared with never users, experimenters were more likely to be out of school and to misuse alcohol – Users were more likely to be male, vocational students or out of school, and to use any of the studied substances (tobacco, alcohol misuse, cannabis)
Wang et al. (31)	– 45,128 students in Hong Kong – Assessment through an anonymous questionnaire	– E-cigarette use was associated with intention to smoke [OR (95% CI)] – In all students [1.74 (1.30–2.31)] – In never-smokers [2.18 (1.12–4.23)] – In ever-smokers [2.79 (2.05–3.79)] – In current smokers, e-cigarette use was significantly associated with [OR (95% CI)] Heavier smoking [2.54 (1.28–3.81)] Morning smoking urge [2.54 (1.50–3.11)] And non-significantly associated with lower quit intention [0.76 (0.52–1.09)] and attempts [0.80 (0.56–1.23)]	– E-cigarette use was associated with intention to smoke. The associations were also significant in experimental and former smokers but not in current smokers – In current smokers, e-cigarette use was significantly associated with heavier smoking and morning smoking, and non-significantly associated with lower quit intention and attempts
Park et al. (32)	– 6,307 middle and high-school students in Korea – Assessment through interview	– Current e-cigarette use [OR (95% CI)] Male gender [3.54 (2.86–4.38)] Higher grade levels (12th school year) [4.06 (1.73–9.52)] Greater average weekly allowance [1.80 (1.36–2.37)] Residence in urban areas [1.37 (1.12–1.69)] Friends’ smoking [3.99 (2.31–6.88)] Daily smoking [2.88 (2.46–3.37)] 10 or more cigarettes smoked per day [3.80 (2.83–5.11)] Attempts to quit smoking [1.52 (1.26–1.82)] At-risk drinking [1.68 (1.41–1.99)] Lifetime drug use or butane gas [2.89 (1.46–5.74)] Lifetime sexual intercourse [1.32 (1.11–1.58)]	– E-cigarette use was associated with male gender, higher grade levels, greater average weekly allowance, residence in urban areas, peers smoking, daily smoking, heavier smoking, and quit attempts – Current e-cigarette use was significantly associated with at-risk drinking, using drugs, and engaging in sexual intercourse
Hughes et al. (33)	– 16,193 middle and high-school students in North West England – Assessment through closed, self-completed – Questions	– Accessed e-cigarettes [OR (95% CI)]s Regular, light smoker [36.55 (28.64–46.64)] Regular, heavy smokers [50.28 (40.97–61.71)] Male gender [1.64 (1.47–1.82)] Having parents/guardians smokers [1.53 (1.37–1.70)] Being occasional, binge drinker [1.46 (1.26–1.69)] Being regular, binge drinker [1.89 (1.59–2.24)]	– E-cigarette access prevalence was highest among smokers, male gender, having parents/guardians smokers, and drinkers
Kinnunen et al. (34)	– 10,233 middle and high-school students in Finland – Assessment through self-administered questionnaires	– Ever-use increased from 17.4 to 25% – Only one-fourth of those who had used e-cigarettes, more than twice, reported quitting smoking as the cause of experimentation – Boys had experimented more often than girls, *P* < 0.001 – E-cigarette ever-use [OR (95% CI)] – Daily cigarette smoking was the strongest determinant [51.75 (38.18–70.14)] – [OR (95% CI)] for e-cigarette use among those who had slightly or much poorer than average academic achievement was [3.30 (2.82–3.87)] in 2013, and [3.84 (2.14–6.91)] in 2015	– E-cigarette ever-use was increasing, and among never-smokers – The most common reason of e-cigarette ever-use was the urge to try something new – Advertisements and parental smoking were associated with e-cigarette experimentation – Daily use was most common among 18 years old boys – Tobacco-related factors were stronger determinants for e-cigarette use than socioeconomic factors – Among socioeconomic characteristics, adolescents’ academic performance was more strongly related to e-cigarette use than family structure, parental education, or working status
Cooper et al. (35)	– 13,602 middle and high-school students in Texas – Assessment through a 38-item anonymous survey, completed either – *Via* paper–pencil or online	– 24.2% of current e-cigarette users had never smoked conventional cigarettes, and 7.3% had never used any other type of tobacco product – Current e-cigarette users [OR (95% CI)] High-school students [74.40 (67.38–80.34)] White [53.17 (43.74–62.37)] Male gender [61.17 (51.51–70.02)] A family member who currently smoked [48.92 (36.53–61.31)] Current cigarette use [48.89 (41.86–55.91)] – Lifetime e-cigarette users [OR (95% CI)] Lifetime cigarette use [36.64 (31.48–41.79)]	– Higher prevalence of current e-cigarette use in high-school students, non-Hispanic white, male gender, and in those who had someone in their home who currently smoked – Both current and lifetime e-cigarette users were also more likely than their peers to use other tobacco products
Fotiou et al. (36)	– 1,320 high-school students in Greece – Assessment through an anonymous self-completed questionnaire	– Six in seven ever e-cigarette smokers had smoked conventional cigarettes – Correlates of dual ever-smoking [OR (95% CI)] Lifetime cannabis use [2.2 (1.4–3.6)] peers who smoke [2.1 (1.2–3.6)] – Lifetime e-cigarette smoking [OR (95% CI)] – Boys [6.8 (3.3–14.0)] – E-cigarettes experimentation [OR (95% CI)] Females’ low-life satisfaction [2.4 (1.2–4.6)]Current conventional tobacco users [2.5 (1.4–5.0)]	– Peers who smoke and lifetime cannabis use were significant correlates of lifetime e-cigarette smoking – Among lifetime smokers, more boys than girls reported e-cigarette use – In lifetime smokers, low-life satisfaction in females and current smoking of conventional tobacco were independently associated with e-cigarettes experimentation
Morean et al. (37)	– 2,241 high-school students in Connecticut – Assessment through an anonymous survey	– All products and the e-cigarette–alcohol class were [OR (95% CI)] More likely to include males {[1.54 (1.03–2.33)], [2.33 (1.47–3.70)], respectively} And less likely to include blacks {[0.28 (0.11–0.73)], [0.09 (0.13–0.58)], respectively}	– The class of students using all products and the e-cigarette–alcohol class were more likely to include males and less likely to include blacks
Larsen et al. (38)	– 6,159 high-school students in Ontario – Assessment through interview and asthma was self-reported	– E-cigarettes smokers [OR (95% CI)] – Asthma [1.78 (1.15–2.76)] – Males [2 (1.37–2.93)] – Students with asthma have a higher odds of smoking e-cigarettes than those without asthma [1.41 (1.04–1.93)]	– Significant factors related to smoking e-cigarettes were boys with asthma – Having doctor diagnosed asthma was significantly associated with a higher odds of smoking any type of substance
Chaffee et al. (39)	– 101,011 middle and high-school students in United States – Assessment through questionnaire	– In 2014 and 2015, past 30-day e-cigarette use exceeded past 30-day cigarette use (in 2015: 9.4% e-cigarettes vs 5.4% cigarettes for females; 13.2% e-cigarettes vs 7.2% cigarettes for males) – The prevalence of using both cigarettes and e-cigarettes at least 1 day in the past month rose 3.7-fold among males	– E-cigarette past month use and ever-use were positively associated with use of cigarettes and other tobacco products – Among male past month cigarette users, there was a positively strong association between past month e-cigarette use and daily cigarette smoking – Past month e-cigarette use among past month cigarette smokers was not associated with cigarette quitting behavior
Miech et al. (40)	– 44,892 middle and high-school students in United States – Assessment through an anonymous questionnaire	– Nicotine prevalence in the past 30 days (% ±SE); vaped nicotine at last use or smoked regular cigarette(s) – 12th grade (12.44% ± 0.71) – 10th grade (7.88% ± 0.55) – Used nicotine vaporizer (% ± SE) – Males (26.29% ± 2.26) vs females (7.53% ± 2.04) – Just flavoring (% ±SE) – Females (69.85 ± 2.40) vs males (61.00 ± 2.23) Hispanics (73.34 ± 3.26) vs non-hispanic white (62.54 ± 2.08)	– In 10th and 12th grades the students with higher frequency of vaping were more likely to vape nicotine rather than vape flavoring – Nicotine was more likely to be vaped by males, by whites, and those who had at least one parent with a college degree – Flavoring was more likely to be vaped by females and by hispanics
Babineau et al. (41)	– 821 high-school students in Ireland – Assessment through an anonymous questionnaire	– 23.8% of participants had used e-cigarettes at least once – Dual trial of traditional and e-cigarettes was common with 69.5% of regular smokers and 30.4% of ever-smokers having tried e-cigarettes – Predictors of continued e-cigarette use [OR (95% Cl)] Females were less likely than males to continue to be e-cigarettes regular users [0.38 (0.16–0.94)]	– A quarter of students reported experimentation with e-cigarettes – Concurrent or experimental use of e-cigarettes and tobacco is more common than sole use, few participants have tried e-cigarettes without having tried conventional cigarettes
Kristjansson et al. (42)	– 6,547 middle school students in United States – Assessment through a national survey	– Family support was significantly stronger for the non-smokers compared with e-cigarettes ever users (OR = 1.033, *P* < 0.001, 95% CI: 1.14–1.55) – Non-smoking participants were more likely than e-cigarettes only users to spend time on homework (*P* = 0.022), and less likely to skip classes (OR = 0.68, *P* < 0.001) or feel alienated from school and studies (*P* < 0.001) – Combustible cigarettes only users were significantly less likely than e-cigarettes users to hang out at a friend’s house without adult attendance (OR = 0.89, *P* = 0.041)	– E-cigarette only users possessed a weaker social support and parental monitoring profile and performed worse in school – E-cigarette only users were more likely to feel alienated from school, to associate with delinquent peers, to spend time outside late at night, and to engage in unsupervised gatherings with their friends
Dautzenberg et al. (43)	– 3,279 middle and high-school students in Paris – Assessment through self-report questionnaire	– E-cigarette experimentation was significantly associated with [OR (95% CI)] Age > 15 years [0.66 (0.46–0.94)] Smoking 10 cigarettes or more [5.67 (3.11–10.34)] Best friends and siblings smoker {[1.54 (1.11–2.14)] and [1.88 (1.41–2.52)], respectively} Experimentation of shisha [2.60 (1.75–3.86)] cannabis use [1.90 (1.32–2.72)] Having two parents who ban smoking [2.32 (1.63–3.30)]	– E-cigarette use was significantly associated with age >15 years, smoking 10 cigarettes or more, best friends and sisters/brothers tobacco use, experimentation of shisha, cannabis use, having two parents who prohibited smoking
Moore et al. (44)	– 10,656 primary and secondary school students in Wales – Assessment through two nationally cross-sectional surveys	– Primary-school students were more prone to have used e-cigarettes (5.8%) compared with tobacco (1.6%) – The proportion of children who had ever smoke e-cigarette and reported currently smoking increased from 6.9% among 10–11 year olds to 39.2% in 15–16 year olds – Current weekly smokers were more likely than non-smokers to report regular e-cigarette use [RRR = 121.15; (95% CI: 57.56–254.97)] – Regular e-cigarette use was more likely among cannabis users [RRR 53.03; (95% CI 38.87–80.65)]	– Primary-school children were more likely to have used e-cigarettes than tobacco – The proportion of children who had ever used an e-cigarette and reported currently smoking increased in higher grades – Current weekly smokers were 100 times more likely than non-smokers to report regular e-cigarette use – Regular e-cigarette use was more likely among those who had used cannabis
Lee et al. (45)	– 6,655 middle and high-school students in South Korean – Assessment through nationally representative cross-sectional survey	– The percentage of frequent e-cigarette users (≥10 days/month) was 3.5% in adolescents who did not smoke within a month, but 28.7% among daily smokers – The most ordinary reason for e-cigarette initiation was curiosity (22.9%), followed by the belief that they are less harmful than traditional cigarettes (18.9%), the motive to cease smoking (13.1%), and the capacity for indoor use (10.7%)	– Frequent and intensive cigarette smoking was associated with frequent e-cigarette users. Frequent users were older – The most common reason for e-cigarette use was curiosity, followed by the belief that they are least harmful than conventional cigarettes, the desire to quit smoking, and the capacity for indoor use
Alcalá et al. (46)	– 1,052 middle and high-school students in California – Assessment through a cross-sectional telephone survey	– E-cigarette use was more common among Ever-smokers of traditional cigarettes compared with never-smokers of traditional cigarettes (47.09 vs 7.54%; *P* < 0.001) Those above 200% of the Federal Poverty Level (13.69 vs 6.77%; *P* < 0.01) US citizens compared with non-citizens (11.44 vs 1.46%; *P* < 0.01) Individuals who spoke English-only compared with those who lived in homes where any language other than English was spoken (13.89 vs 6.76%; *P* < 0.05; OR = 2.22)	– E-cigarette use was more common among ever-smokers of traditional cigarettes, individuals above 200% of the Federal Poverty Level, US citizens and those who spoke English-only – Citizenship status and language spoken at home were associated with lifetime e-cigarette use
Wills et al. (47)	– 2,309 high-school students in Hawaii – Assessment through a cross-sectional survey	– Non-smokers who had used e-cigarettes displayed more willingness to smoke cigarettes compared with those who had never used any tobacco product [OR 2.35 (95% Cl: 1.73–3.19)]	– Non-smokers who had used e-cigarettes presented more willingness to smoke cigarettes compared with non-smokers – Willingness predicted smoking onset

Characteristics of vulnerable adolescent populations:

• *Male gender*: Gender was found to be a predictor of current e-cigarette use with males being significantly more likely to declare having already tried it than females (6, 20, 21, 24, 29–41).
• *School performance*: Vocational school career (29, 30), lower school performance (29, 34, 42), being out of school (30), and studying at disadvantaged school (30) have been demonstrated that are correlated with both e-cigarette ever- and daily use. These factors are also predisposing to conventional smoking (43). On the other hand, in a recent study, researchers have found that non-users and e-cigarette-only users had higher mean grades than the cigarette-only group, and the non-user group had higher mean grades than the dual user group (20).
• *Age-grade*: In a previous study, both increased use of e-cigarette, and e-cigarette perceived harmfulness and awareness have been delineated with advancing school grade (23). This, steady with age, increase has been demonstrated in several studies (20, 22, 29, 32, 38, 40, 43–45), highlighting the urgent need of novel research to shed further light into the age-related trajectories of e-cigarette use.
• *Economic status*: Both being in employment and being affluent (29, 32, 46) provide higher odds of using e-cigarettes.
• *Tobacco use and related factors*: Tobacco-related determinants are stronger characteristics of e-cigarettes usage than sociodemographic factors (34). Daily smoking (6, 24, 29, 30, 32–34, 39, 43, 44, 46), parental or household member smoking (29, 33–35, 43), peers smoking (20, 29, 30, 32, 36, 43, 44), ever-use of all tobacco products like snus, and waterpipes (20, 29, 34, 39, 44) were associated with e-cigarette use.

## Discussion

As most studies report, male gender, older age, higher amount of pocket money, and tobacco smoking-related characteristics, such as regular and heavier smoking, and having peers who smoke, are the most common trends in characteristics of adolescent e-cigarette users.

The increased prevalence of vaping among males can be due to sociocultural characteristics or marketing messages and current trends. In many cases, males are most likely to be early adopters of technology, having easier access to e-cigarettes and they can also get exposed to e-cigarettes because they represent a newcomer product (29, 33). Additionally, it was demonstrated that boys’ higher risk of e-cigarette use may exist, partially, due to their lower harm perception (48). Generally, males tend to appraise lower risk comparative with females and stay away of risky behaviors only when they perceive severe risk (49).

One might say that the observed frequent use of e-cigarettes in older age is expected since older students are more informed about e-cigarettes, and e-cigarettes are easier to obtain from retail shops and through the internet (19, 44), given the lack of regulation of age restrictions laws, a situation which is recently being reviewed in numerous countries (27, 50).

Another characteristic, the observed relationship between higher amount of pocket money and e-cigarette usage may be due to the fact that adolescents can afford to buy e-cigarettes (51). This characteristic may suggest that having an adequate allowance at the adolescent’s disposal may influence smoking practice, suggesting that guardians, who provide youths with pocket money, should pay attention in how that cash is spend.

Several studies also support the association between vaping and tobacco use related characteristics. Indeed, it has been proposed that e-cigarettes can be used as a method for smoking cessation. However, previous research has shown that vaping among adolescents was faced more for experimentation rather than smoking cessation (33), and that heavier e-cigarettes smokers are least likely to consider smoking cessation (29). A great percentage of young vapers had never tried conventional cigarettes (29, 30, 33, 35, 44), while in other studies e-cigarette ever-use was non-significantly related neither with quit intention nor attempts (24, 39). On the contrary, among adults, e-cigarettes are seen as a potential cessation aid (11, 52), while among adolescents who have never before smoked, e-cigarette use is associated with willingness to smoke, and vaping may act as a “one-way bridge” to smoking (47, 53). Moreover, an association was recently reported between e-cigarette use and initiation or escalation of cigarette smoking (28). Only in two Korean studies, e-cigarette usage was associated with the desire to quit smoking (32, 45).

A number of limitations of studies included should be considered in order to allow interpretation of the described findings. Firstly, the cross-sectional design in many studies preclude us from exporting causal inferences about the results; since they can only indicate associations among the studied characteristics but not causality (6, 20, 24, 29–33, 35, 36, 38, 39, 41–47). Secondly, the self-reported assessment could introduce reporting bias (6, 20–24, 29–46, 52). Thirdly, findings may not be generalizable to populations outside of the samples geographical areas or other countries or regions (20, 21, 23, 29, 30, 32–38, 41–47).

It is urgent, moreover, to include e-cigarettes in tobacco prevention programs; targeting in vulnerable groups through early intervention efforts. Given their overwhelming acceptance, prevention campaigns *via* social media, appear to be an effective mechanism for influencing trends when targeting youth populations. Prospective surveys should be directed toward addressing the potential long-term effects on health and the probable nicotine addiction of consumers. The findings underscore the need of constructing persuasive e-cigarette prevention messages promoting public health welfare.

## Author Contributions

EPP contributed in designing and drafting the manuscript, PS contributed in the initial conception and critical revision, EP contributed in the design and interpretation, TC contributed in the interpretation and critical revision, EN contributed in the conception, interpretation, and critical revision. All authors provide their approval for the final version to be published.

## Conflict of Interest Statement

The authors declare that the research was conducted in the absence of any commercial or financial relationships that could be construed as a potential conflict of interest.
